# Predictors of Weight Loss and Weight Gain in Weight Management Patients during the COVID-19 Pandemic

**DOI:** 10.1155/2021/4881430

**Published:** 2021-12-17

**Authors:** Jennifer L. Kuk, Rebecca A. G. Christensen, Elham Kamran Samani, Sean Wharton

**Affiliations:** ^1^School of Kinesiology and Health Science, York University, Toronto, Canada; ^2^The Wharton Medical Clinic, Hamilton, Canada; ^3^Dalla Lana School of Public Health, University of Toronto, Toronto, Canada

## Abstract

**Objective:**

To examine the associations between patient struggles, health, and weight management changes during the COVID-19 pandemic.

**Methods:**

585 patients attending a publicly funded clinical weight management program responded to an electronic survey.

**Results:**

Over half of the patients reported worsened overall health, mental health, physical activity, or diet during the pandemic. Approximately 30% of patients lost ≥3% of their body weight and 21% gained ≥3% of their body weight between March and July of the pandemic. Reports of social isolation was associated with increased odds for weight loss in women (OR = 2.0, 1.2–3.3), while low motivation (OR = 1.9, 1.0–3.7), depression (OR = 2.5, 1.0–6.3), and struggles with carbohydrate intake (OR = 2.1, 1.0–4.3) were associated with weight gain. Cooking more at home/eating less take out was associated with increased likelihood of weight loss (OR = 2.1, 1.1–3.9) and lower odds for weight gain (OR = 0.2, 0.1 to 0.97). Working from home was not associated with weight loss or weight gain (*P* > 0.6).

**Conclusion:**

The COVID-19 pandemic is associated with certain factors that may facilitate weight loss and other factors that promote weight gain. Thus, depending on the patient experience during the pandemic, prevention of weight gain may be more appropriate than weight loss.

## 1. Introduction

With the COVID-19 pandemic there have been many governmental restrictions imposed that may have a dramatic impact on weight management. Most of the literature has reported weight gain in the general population [1, 2]. However, the average response may mask differences within subgroups in the population. Seal et al. [3] report that weight gains of greater than 2 kg are more likely in those with obesity, but it is unclear if this also applies to populations who may be attempting weight management. In particular, there may be differences in those who are focused on obesity management in their response to the same governmental restrictions that may result in positive or negative obesity management effects. For example, closures to fitness facilities and stay-at-home orders may have negatively impacted physical activity [4]. Conversely, the increased rates of working from home means that the lack of commuting may have freed up more time to be able to physically active. Changes in physical activity are associated with weight change during the COVID-19 pandemic stay-at-home mandates [3, 5]. Similarly, the stay-at-home orders may have resulted in individuals cooking and eating more reasonable portion sizes instead of eating out or take-out [4, 6]. Conversely, the stay-at-home order may make grocery shopping and access to healthy foods more difficult [6]. Further, patients may find that continuous access to food and snacks may increase caloric intake [6]. Increased intake of snacks and food consumption is reported to be associated with greater weight gain [7, 8]. With the pandemic, there has also been increased stress [2, 7], loss of employment [2], and less access to healthcare and social isolation, all of which may influence weight management. The majority of the research to date has focused on weight gain and negative health consequences of COVID-19 in the general population [1, 2], which may differ from correlates of weight loss, particularly in a sample that is more focused on weight management.

Thus, the objective of this study is to describe the changes in self-reported health and weight management with the pandemic for patients enrolling in a publicly funded evidence-based clinical obesity management program. Further, we aim to examine the association between social factors during the pandemic with weight gain or weight loss.

## 2. Materials and Methods

### 2.1. Study Population

There were 1254 patients from the Wharton Medical Clinic (WMC) Weight Management Program who responded to an electronic survey between July 2020 and January 2021 evaluating the clinic program and assessing pandemic weight management. Of these, 730 participants gave their written consent for the use of their medical data for research purposes. Patients were informed that their decision to participate would not influence their medical treatment at WMC. Participants did not receive any form of stipends. All methods were approved by the York University Research Ethics Board (Ethics Certificate #: 2009–117, 2013–123, e2017–166).

As described previously [9–11], WMC is a physician referral-based clinic designed to educate and enable patients to implement behavioural, pharmacological, or surgical strategies to manage their weight and improve their health. Patients attend the clinic on a monthly basis or as needed to have individual meetings with bariatric educators to discuss personalized weight management strategies, dietary plans, and physical activity options. Patients meet with physicians to discuss medication options, interest in bariatric surgery, and any obesity related comorbidities. If indicated, patients are referred for additional tests, to other medical professionals or for bariatric surgery. Since March 17, 2020, of the pandemic, weight management services have transitioned to virtual visits via telephone. Patients can attend the program for as long as they wish and are able to return to the clinic after long absences. The clinic operates within the Ontario Health Insurance Plan and all services are provided at no charge to the patient.

An invitation to participate in an electronic questionnaire was sent to patients. The questionnaire asked participants about weight management behaviours during the pandemic, their satisfaction with the clinic care during the pandemic, suggestions for improvement to care, and physical health, mental health, employment, social, and other factors related to the pandemic. Specifically, patients were asked, “The COVID-19 pandemic has had the following effect on my mental health: negative effect, No effect or Positive effect.” “Since the COVID-19 pandemic: I feel less healthy, My health has remained the same or My health has improved.” “The COVID-19 pandemic has had the following effect on my eating: Worsened, No effect or Improved.” “My activity levels during the COVID-19 pandemic have: Decreased, Stayed the same or Increased;” “Since the COVID-19 pandemic have you found engaging in weight management behaviours: much harder, Harder, No different, Easier or Much Easier.” Patients were asked two open-ended questions: “Please tell us about your weight management experience during the pandemic.” And “what have you struggled with during the COVID-19 pandemic?” (176 participants did not respond to this question). Categories for the common responses for struggles during the pandemic were created as follows: work, family care, travel restrictions/home bound, physical activity, lack/low energy, diet, carbohydrate intake, stress, stress/anxiety related to COVID-19, social isolation/loneliness, sleep, mental health, depression, lack/low motivation, health/access to healthcare, income, and boredom (each dummy coded as yes/no). Participants that reported that their diet had worsened or that they struggled with diet were categorized as struggling with diet. Similarly, participants that reported that their physical activity had decreased or that they struggled with exercise or physical activity were categorized as struggling with physical activity. Participants also reported their weight on the first of March and July 2020 during the pandemic. Patients reporting a body weight less than 50 kg were excluded from the analyses. Of the 730 participants, 585 had complete data on their weight change during the pandemic and were included in this analysis.

Weight change was calculated as the difference between the July and March body weights, and percent changes were calculated relative to the March body weight. Participants with greater than 3% weight loss were classified as having lost weight, and those with greater than 3% weight gain were classified as having gained weight.

### 2.2. Statistical Analyses

Patient characteristics are presented as means (SD) for continuous variables and frequency and prevalence (*n*, %) for categorical variables. Sex differences in patient characteristics, struggles, and behaviors were assessed with *t*-tests and chi-square tests. Differences in diet or physical activity-related struggles and other COVID-issues (social isolation, stress, working from home, mental health, and physical health) were examined with chi-square tests. Associations between each of the COVID-19 struggles and the odds ratio for weight gain or weight loss were assessed separately using logistic regression with examination for gender by struggle interaction and main effects adjusting for age and body weight in March. All statistical analyses were conducted using SAS version 9.4 (SAS Institute, Cary, NC, USA). Statistical significance was established at *P* < 0.05.

## 3. Results

Participant characteristics are shown in Table 1. Patients were predominately female and had a mean age of 54 ± 12.8 years and a wide range in self-reported baseline body weight (53 to 209 kg). Thirty-one percent of women and 28.6% of men reported losing weight at least 3% of their body weight, while 21% of women and 22% of men gained at least 3% of their body weight between March and July. Two-thirds of the patients reported being worried or very worried about the COVID-19 pandemic.

**Figure 1:**
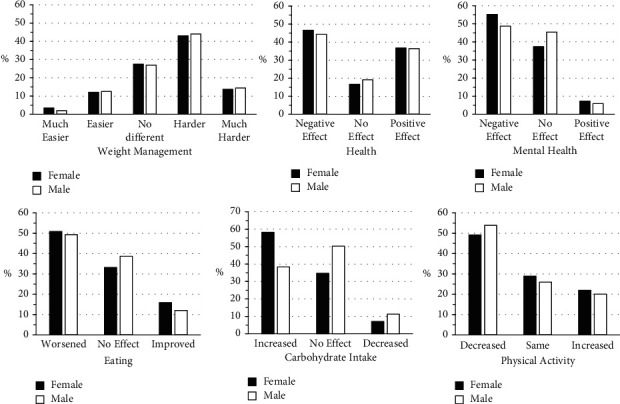
Changes in weight management, health, mental health, eating, carbohydrate intake, and physical activity with the COVID-pandemic. No sex differences (*P* > 0.05).

Fifty-seven percent of patients report that weight management was harder or much harder since the COVID-19 pandemic, with 16% of patients finding it easier or much easier with no sex differences (*P*=0.92, Figure 1). Three percent of patients report that they were not attempting to manage their obesity during the pandemic. Approximately half of the patients self-reported that the pandemic had a negative effect on their health, mental health, eating, and physical activity. When factors were examined together, over ∼60% of patients reported that their physical and/or mental health have worsened with the pandemic, and 74% of females and 79% of males reported that their diet and/or physical activity have worsened with the pandemic, while only 7.5% of females and 4% of males report that both their diet and physical activity had improved with the pandemic. A small proportion of patients reported that they had increased their frequency of cooking at home/reduced the frequency of eating out or ordering take-out (female: 8%, males: 6%).

**Figure 2:**
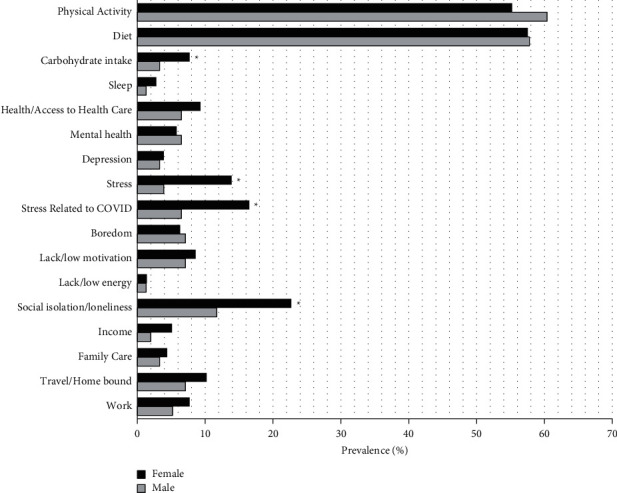
Sex differences in the self-reported struggles with the COVID-19 pandemic. ^*∗*^Significantly different from males (*P* < 0.05).

Figure 2 demonstrates the struggles patients self-reported experiencing during the COVID-19 pandemic. Seventy-seven percent of patients report having some sort of struggle with weight management during the pandemic. Struggles with or a worsening diet or physical activity were reported by over half of males and females. Females were more likely to report experiences of social isolation or loneliness, stress or stress specifically related to COVID, and struggles with carbohydrate intake than males (*P* < 0.05).

When the factors were examined together, men and women with worsened physical and mental health were associated with more diet struggles than those who did not (71.6–82.3% versus 39.6–45.0%, *P* < 0.001). Similarly, men and women with worsened health were more likely to have struggles with physical activity (78.5–87.3% versus 41.8–45.5%, *P* < 0.0001), while mental health was associated with worsened physical activity in females (62.4% versus 46.7%, *P*=0.001) and not men (64.9% versus 56.3%, *P*=0.28). Patients reporting struggles with social isolation were less likely to also struggle with physical activity (M: 39% versus 63%; F: 43% versus 59%, *P* < 0.05) and diet (female only: 42% versus 62%, *P*=0.0003) than those who did not report issues with social isolation. There were no differences in diet or physical activity between those working at home versus those who were not (*P* > 0.4). Patients reporting stress were more likely to report struggles with diet (74% versus 55%, *P*=0.004—males and females combined due to small sample size), but not physical activity (*P*=0.16).

Table 2 reports the odds ratios for experiencing a ≥3% weight loss or weight gain between March and July 2020 during the COVID-19 pandemic with struggles experienced weight examination for gender interaction effects, adjusting for age and weight in March. There were significant gender interaction effects for struggles with social isolation and weight loss, so the odds ratios were presented for males and females separately (*P*=0.02). Specifically, the association with social isolation was only significant in females, wherein social isolation was associated with a greater odds of weight loss (OR = 2.0, 1.2–3.2). In the sample with men and women combined, struggles with physical activity (OR = 0.47, 0.3–0.7) and diet (OR = 0.32, 0.2–0.5) were associated with a lower odds for weight loss, while being homebound/having travel restrictions (OR = 2.0, 1.1–3.6) and cooking more at home/eating less take out was associated with increased likelihood of weight loss (OR = 2.1, 1.1–3.9, *P*=0.02).

Struggles with exercise (OR = 2.7, 1.7–4.2), diet (OR = 8.6, 4.8–15.6), carbohydrate intake (OR = 2.1, 1.0–4.3), depression (OR = 2.5, 2.0–6.3), and low motivation (OR = 1.9, 1.0–3.7) were significantly associated with higher odds of weight gain (*P* < 0.01) adjusting for age, gender, and body weight in March. Cooking more at home/eating less take out was associated with a lower odds of weight gain (OR = 0.17, 0.04 to 0.69). Working from home was not associated with weight loss or weight gain (*P* > 0.4).

## 4. Discussion

Our unique study provides insight into the obesity management and health struggles experienced by patients during the COVID-19 pandemic. Over half of the patients report worsening overall health and mental health and worsened physical activity or diet during the pandemic. Difficulties with physical activity and diet were associated with increased odds of weight gain and a lower odds of weight loss. Struggles with social isolation was associated with increased odds for weight loss in only women, while struggles with carbohydrate intake, low motivation, and depression were associated with weight gain in both men and women. Thus, the COVID-19 pandemic is associated with factors that can facilitate weight loss and promote weight gain in different patients.

Factors that can impact obesity management and health are numerous, and for most, COVID-19 has caused uncertainty and many changes to the way people live their lives and their ability to manage their health and obesity, such as limiting of social interaction, working from home/loss of employment, closures of fitness facilities, limiting/banning in-restaurant dining, less frequent trips to the grocery store, and changes to access to health care [4, 6]. Within our sample, over 50% of patients report that obesity management has been harder with the COVID-19 pandemic, while ∼15% report that it has been easier and 3% report that they were not engaging in weight management. Over this time period, ∼30% of obesity management patients were able to lose 3% or more weight of their body weight during the pandemic, with 21% gaining ≥3% of their body weight. As a comparison, our unpublished data suggests that ∼45% of patients were about to lose 3% of their body weight or more over a 3-month period, and only 3.5% of patients gain over 3% of their body weight within 3 months of enrolling at the clinic. Thus, there appears to be a general trend for worsened obesity management during the pandemic.

Obesity is associated with greater weight gain during the pandemic, but also greater rates of weight loss than normal weight individuals [8]. However, as compared to individuals living in France during the lockdown with overweight/obesity and diabetes [8], these Canadian weight management patients were less likely to gain weight and more likely to lose weight. However, this may not be surprising as not all individuals with obesity may be concerned with preventing weight gain or attempting weight loss; thus intentions may be an important consideration to examine. In participants actively recruited to a biweekly 10-week weight loss intervention offered on Zoom, there was a substantively larger weight loss reported than what was observed in this study (5.8 versus 1.3 kg) [12]. Our study included patients that were in various points of their weight management care, and so they could be in the early active weight loss phase or in the later weight maintenance phase. Further, this weight management sample included people who voiced that their weight management has now become secondary to the pressures of COVID-19. This may reflect the divergent responses of individuals in response to the pandemic lockdowns in that some may be able to better succeed at weight management while others struggle.

For most patients, the pandemic has had significantly negative impact on health. Over half of patients report worsening physical and mental health with the pandemic. Specifically, approximately three quarters of patients report that their eating and/or physical activity has worsened with the pandemic. While the changes in physical activity are similar to what is reported in a nonclinical sample of French adults with overweight/obesity [8], changes to dietary intake were worse in our clinical sample. In this study, working from home was not significantly associated with diet or physical activity struggles. There appears to be a dichotomy in diet and physical activity responses to home isolation that may be related to a number of factors. For example, working at home was reported to give some patients more time to be active as they no longer needed to commute, while others cited that their activity options were limited at home and with the fitness facility closures. Similarly, some participants commented that it was easier to regulate their eating when they are at home and did not eat out, while others reported that they struggled with eating with the close proximity to their kitchen and snacks or having to cater to the dietary preferences of others in their household. These divergent responses may be why we and others [13] report that working from home is not associated with weight gain. The less frequent trips to the grocery store for some patients meant more limited fresh produce while others found this helpful for their meal planning and better overall eating habits. We observed that worse physical and mental health and low motivation were all associated with worsened diet and physical activity. Similar to another study [7], this study indicates that stress is associated with diet struggles. Stress is well-established to be linked with increased food consumption and reports during COVID-have shown similar findings with weight gain being more prevalent in those reporting weight gain [2]. Further research is needed to unpack how these factors interrelate and impact lifestyle behaviours and weight management or if reverse causation may be at play.

A modest proportion of patients cited low motivation during the pandemic. Motivation is commonly thought to be critical for weight loss success [14], but not all studies demonstrate that motivation is associated with weight loss [15]. This may be because autonomous motivation (intrinsic goals or self-directed), as opposed to controlled motivation (external reward or fear of punishment) may be more important for health behaviours [16–18]. Low motivation was associated with a higher prevalence of diet and physical activity struggles but was only associated with weight gain and not weight loss in our study. In this study, we did not assess the specific types of motivation or which aspect of weight management the motivation was related to, but the lack of association may also reflect the strong physiological processes that defend against weight loss independent of personal choice and motivation [19]. Further research is needed to confirm the popularly held belief that motivation is needed for weight loss.

Interestingly, we observe that social isolation was associated with fewer diet and physical activity struggles in women and less physical activity struggles in men. Social isolation was also associated with an increased likelihood of losing weight in women, but not men. Social support is an integral part of several obesity management programs and is associated with better dietary adherence [20], better weight management [21], and even lower mortality risk [22]. Family and friends are the most commonly reported sources of social support for weight loss among primary care weight management patients, with women receiving more support than men [23]. Thus, it is unclear why social isolation during the pandemic was associated with positive effects on weight management and lifestyle behaviours. However, social interaction may not be the same as social support. Marquez et al. [24] report that success for Latin women in a lifestyle weight management program may be associated with the weight change and weight status of their close social ties. Given that weight gain is associated with the weight gain of one's close social network [25], isolation may be protecting patients from some of the negative influences of their social network, particularly for women. Some patients reported that the pandemic did not allow them to have their regular outings with eating and alcohol consumption. Alcohol consumption has increased during the COVID-19 lockdown [1], which may impair weight loss efforts [8]. Additionally, the type of support may also be important in determining whether the support is helpful for weight management [26]. Specifically, complements and offers to engage in weight management with the individual was associated with better weight management, while those who regained weight are more likely to receive reminders and suggestions from their support network [26]. Clearly, more work is needed to understand the impacts of the pandemic and social support on lifestyle behaviours and obesity management and the potential sex differences that may exist.

We and others [8, 27] observe that worsened diet and physical activity were both strongly associated with increased risk for weight gain and lower odds for weight loss. In accordance with previous literature, cooking at home and eating less takeout was also associated with better weight management [28]. However, while social isolation was associated with weight loss in women, it was not associated with weight gain. Similarly, depression, motivation, and struggles with carbohydrate intake were associated with only increased risk for weight gain and not weight loss. Thus, factors that are associated with weight loss may not necessarily be the same factors that are associated with weight gain. Traditionally, similar dietary, physical activity, stress, and behaviour modification strategies are targeted for weight gain prevention and weight loss interventions [19, 29], with additional pharmacotherapy and surgical interventions reserved for weight loss [19]. However, given that the factors that are associated with weight loss and weight gain may differ, a refinement or adaptation of weight management goals may be necessary depending on the patient situation. For example, patients struggling with low motivation during the pandemic may wish to target prevention of weight gain as opposed to weight loss.

Strengths and limitations of the current study warrant mention. Our study is a retrospective analysis of an opportunistic dataset from sample of predominately white, middle-aged female patients who responded to an electronic questionnaire aimed at assessing changes in patient needs with the pandemic and receiving patient evaluation and comments regarding virtual visits. Thus, the applicability of these findings to other demographics is unclear. Further, patients responded to an open-ended question regarding their struggles and experiences during the pandemic and did not specifically respond to questions regarding whether they had struggles with motivation, sleep, stress, boredom, and so on. Thus, the prevalence of these struggles is likely underestimated. Nevertheless, these responses are likely reflective of the most significant or top of mind struggles experienced during the pandemic. Further, patients were not asked specifically about pharmaceutical or bariatric surgical interventions during the pandemic; however, no patients mentioned these interventions when asked: “Please tell us about your weight management experience during the pandemic.” This analysis is also based on patient recall of past weight loss over the pandemic and is subject to bias and error in reporting [30]. However, it has been reported that populations who are attempting weight loss may be more accurate with their self-reported body weights [31, 32].

Thus, we observe that half of the patients attending a publicly funded weight management clinic have had negative effects on their physical and mental health, and their eating and physical activity behaviours over the pandemic. Some patients had positive effects on weight change, correlating to reports of social isolation. We also observed that factors associated with weight loss and weight gain may differ and that there may be sex differences in how the pandemic has affected weight management.

## Figures and Tables

**Table 1:** Participant characteristics.

	Women	Men
*N*	431	154
Age	54.0 (12.8)	54.2 (13.5)
March weight (kg)	97.5 (22.3)	116.3 (26.8)^*∗*^
July weight (kg)	96.2 (21.6)	116.0 (27.0)^*∗*^
Weight change (kg)	−1.3 (6.4)	−0.3 (8.5)
% weight change	−1.0 (6.3)	0.0 (8.9)
% lost 3% body weight	30.9%	28.6%
% gained 3% body weight	20.9%	22.1%
Worry about COVID-(*n*, %)		
Verry worried	127, 29.5%	39, 25.3%
Worried	155, 36.0%	69, 44.8%
Somewhat worried	127, 29.5%	33, 21.4%
Not worried at all	22, 5.1%	13, 8.4%
Work at home during COVID-19	108, 39.6%	52, 54.7%^*∗*^
Work at home before COVID-19	28, 10.6%	19, 19.0%^*∗*^
Employment negatively affected by COVID-19	97, 36.5%	37, 36.3%
Wears a mask all the time when going out	382, 88.8%	115, 74.7%^*∗*^

^*∗*^Significant sex difference (*P* < 0.05).

**Table 2:** Associations between the odds of weight loss or weight gain with self-reported COVID-19 struggles.

	Weight loss			Weight gain		
	OR	95% CI	*P* value	OR	95% CI	*P* value
Health	0.88	0.5–1.7	0.71	0.58	0.3–1.3	0.20
Mental health	1.05	0.5–2.2	0.91	1.25	0.6–2.8	0.59
Mental/social						
Depression	0.41	0.1–1.3	0.12	2.51	1.0–6.3	0.049
Stress	0.77	0.4–1.4	0.38	1.16	0.6–2.2	0.65
COVID-related stress	0.64	0.4–1.1	0.11	0.77	0.4–1.4	0.41
Boredom	0.60	0.3–1.4	0.22	1.20	0.5–2.7	0.66
Low motivation	0.69	0.3–1.4	0.30	1.94	1.0–3.7	0.04
Low energy	0.52	0.1–4.4	0.55	0.52	0.1–4.4	0.55
Social isolation/loneliness	M: 0.32 F: 2.01	0.1–1.5 1.2–3.3	0.15 0.005	0.95	0.6–1.6	0.85
Lifestyle						
Exercise	0.47	0.3–0.7	<0.0001	2.70	1.7–4.2	<0.0001
Diet	0.32	0.2–0.5	<0.0001	8.63	4.8–15.6	<0.0001
Carbohydrate intake	0.90	0.4–1.9	0.78	2.08	1.0–4.3	0.05
Sleep	0.45	0.1–2.1	0.31	0.58	0.1–2.6	0.48
Other						
Income	0.97	0.4–2.3	0.94	1.20	0.5–3.1	0.71
Family care	1.33	0.6–3.2	0.52	1.20	0.5–3.1	0.71
Travel/homebound	2.02	1.1–3.6	0.02	0.88	0.4–1.8	0.73
Work	0.83	0.4–1.4	0.62	0.68	0.3–1.6	0.37

Models with significant gender interaction effects have odd ratios for men and women presented separately. Models for each struggle were examined separately and adjusted for age, gender, and body weight in March.

## Data Availability

The datasets generated and analyzed during the current study are not publicly available due to privacy laws associated with medical data but are available with a data sharing agreement as approved by the relevant institutional ethics committee and the health information custodian (S. Wharton).
